# Bayesian clustering of 109 worldwide countries according to the trend of prostate cancer mortality rates from 1990 to 2019

**DOI:** 10.1371/journal.pone.0290110

**Published:** 2023-08-14

**Authors:** Tofigh Mobaderi, Anoshirvan Kazemnejad, Masoud Salehi

**Affiliations:** 1 Department of Biostatistics, Faculty of Medical Sciences, Tarbiat Modares University, Tehran, Iran; 2 Department of Biostatistics, School of Public Health, Iran University of Medical Sciences, Tehran, Iran; Chung Shan Medical University, TAIWAN

## Abstract

Neglecting possible growth heterogeneity and focusing only on the overall pattern of prostate cancer mortality rates can result in misunderstandings and incorrect conclusions about the growth process of the outcome. The main goal of this study was to capture the heterogeneity of prostate cancer mortality rates among countries from 1990 to 2019. To accomplish this aim, we performed the Bayesian latent Growth Mixture Model (GMM). In this longitudinal study, Prostate cancer mortality rates data from 1990 to 2019, as well as the Human Development Index (HDI), the Gross National Income (GNI), and the Life Expectancy at Birth (LEB), were obtained from WHO and UNDP platforms. The Bayesian GMM was used to discover homogeneous subgroups and estimate the pattern of prostate cancer mortality rates in each subgroup. The HDI, GNI and life expectancy effects were estimated using a Bayesian conditional Latent Growth Model (LGM). Globally, the intercept and the slope of the Bayesian LGM were equal to 8.77 and 0.19, respectively. The Bayesian GMM classified the 109 countries into four groups, which had significant positive growth patterns with different slopes except for the first class. The effect of HDI was not significant on the overall prostate cancer death rates, but GNI and LEB had a significantly positive effect on the model’s intercepts and a significantly negative effect on the slope. Although the prostate cancer mortality rate increased globally, it has four distinct latent subgroups with various patterns. Additionally, the effects of HDI, GNI, and LEB on prostate cancer mortality rates varied significantly among the four subgroups, indicating a need for further investigation to identify causal factors.

## Introduction

Prostate cancer is a challenging disease that represents a major cause of illness and mortality among men and has a significant global impact on millions of individuals [1]. It is the second most commonly diagnosed cancer in men globally, accounting for 7% of all newly diagnosed cancer cases in men [2]. Annually, over 1.4 million new cases of prostate cancer are diagnosed, and in 2020, the number of deaths resulting from prostate cancer exceeded 370,000 worldwide [3].

Previous researches show the occurrence of prostate cancer worldwide is positively associated with the Human Development Index (HDI) and Gross Domestic Product (GDP), which typically results in higher incidence rates in developed countries compared to less developed countries [4, 5]. The areas with the highest incidence of prostate cancer are Australia and New Zealand in Oceania, North America, Western and Northern Europe, and the Caribbean [6]. Conversely, regions containing numerous low-income nations, including Central Asia, South Asia, and sub-Saharan Africa, presently have the lowest incidence rates of prostate cancer [1]. Asian countries with a high HDI, such as Japan and South Korea, had lower incidence rates of prostate cancer than Western countries with similar HDIs [7]. Nevertheless, prostate cancer incidence has increased in these areas in recent years [8]. In terms of mortality rates, the Caribbean nations of Barbados, Trinidad and Tobago, and Cuba, the sub-Saharan African country of South Africa, and former Soviet Union states of Lithuania, Estonia and Latvia showed the highest rates. On the other hand, Thailand and Turkmenistan in Asia had the lowest mortality rates [6]. Variations in the patterns of prostate cancer mortality rates among countries may be related to HDI, GDP, life expectancy, ethnicity, Prostate-Specific Antigen (PSA) screening, lifestyle choices, dietary habits, and environmental exposure [5, 6, 9–11].

Analyzing the patterns of growth in prostate cancer mortality rates over time can offer valuable insights for policymakers to identify and investigate risk factors associated with the disease. There are several statistical techniques that can be utilized to evaluate trends over time. Latent Growth Models (LGM) are a more generalized approach that allows for the inclusion of latent variables in modeling and can handle multiple outcome growth processes [12]. However, it’s important to note that this method assumes that all individuals in the sample belong to a single homogeneous population and that their individual growth trajectories vary randomly around the overall mean growth trajectory [13]. Neglecting possible growth heterogeneity and focusing only on the overall mean growth trajectory can result in misunderstanding and incorrect conclusions about outcome growth [14]. In recent years, the latent Growth Mixture Models (GMM) have become increasingly popular in the analysis of longitudinal data. GMM is an extension of LGM that allows for the presence of population heterogeneity in the growth of outcomes by grouping individual trajectories into clusters or classes [14]. In other words, GMM identifies homogeneous latent groups of individuals with distinct growth trajectories as well as estimates the trend of each group. However, due to the complexity of the GMM model and sample size restrictions, the Maximum Likelihood Ratio (MLR) approach often fails to estimate the parameters. As a result, the Bayesian approach could be used to overcome this issue.

Based on our literature review, we couldn’t find published research that evaluates the patterns of prostate cancer mortality rates over the past few decades while accounting for the heterogeneity among countries worldwide. Hence, the objective of the current study was to cluster countries into homogeneous subgroups based on the prostate cancer mortality rates pattern by implementing Bayesian GMM on the World Health Organization (WHO) data and using Global Barden of Disease (GBD) dataset as prior information. The findings of the investigation may help to improve our understanding of the trends in prostate cancer mortality rates, and the categorization of countries could provide insights into potential risk factors and guide public health interventions.

## Materials and methods

In this longitudinal study, data was obtained from three international sources—World Health Organization (WHO), Global Burden of Disease (GBD), and United Nations Development Programme (UNDP). The prostate cancer mortality rates data per 100,000 men was obtained from the WHO platform which included 111 countries and territories with thirty time points from 1990 to 2019 [15]. However, two countries were excluded due to missing data. The final dataset included 109 countries, where prostate cancer mortality rates were reported at least twice for each country. The GBD platform was used to extract estimated prostate cancer mortality rates for the same regions from 1990 to 2019 [16]. The information of 104 out of 109 regions was available in GBD which was compatible with WHO dataset. This GBD data was utilized to set informative priors for the Bayesian analysis. The UNDP platform was utilized to extract data on three different covariates: the Human Development Index (HDI), The Gross National Income (GNI) per capita, and The Life Expectancy at Birth (LEB) for each country from 1990 to 2019 [17]. The arithmetic mean of each variable was computed for every country throughout the study period. The HDI is expressed as a value between 0 and 1, the GNI per capita is the dollar value of a country’s final income in a year divided by its population using Atlas methodology, and the LEB indicates the number of years a newborn infant would live if prevailing patterns of mortality at the time of its birth were to stay the same throughout its life [18, 19]. In this study, the GNI was divided by 1000 dollars, so each unit of GNI was equivalent to 1000 dollars.

**Fig 1:**
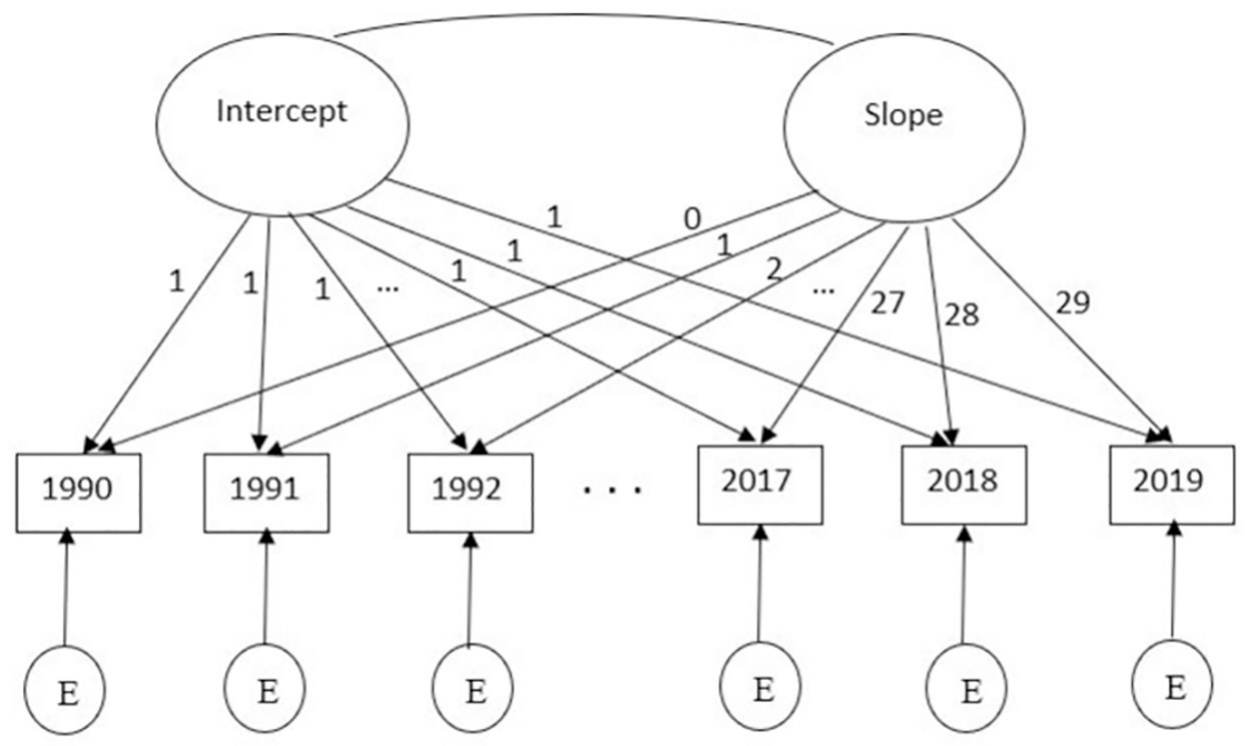
The conceptual diagram of the unconditional linear LGM.

Latent Growth Models (LGM) are a specific type of Structural Equation Modeling (SEM) that utilize two latent components, known as intercept and slope, to estimate the patterns of a response variable in longitudinal study [20]. The conceptual diagram of simple linear LGM without covariates i.e., unconditional model is depicted in Fig 1.

In Fig 1, the ovals represent the latent intercept and slope factors, which indicate the initial level of the outcome measure and the rate of outcome change over time, respectively [13]. The factor loadings on the intercept are fixed at 1.0 to represent the mean of the response variable at the first time point. In contrast, the factor loadings on the slope determining the form of the growth process, whether it is linear or nonlinear. The equal intervals between the factor loadings on the slope indicate a linear growth process. Previous studies have suggested that the growth process of prostate cancer mortality rate was approximately linear [6, 11, 21]. Therefore, in this study, the growth process is considered to be linear, and the factor loadings on the slope are set to increase by one unit at each time point from 0 to 29.

Due to restrictions on using the Maximum Likelihood Ratio (MLR) method for small sample sizes, particularly for clusters, the Bayesian approach was used to estimate the parameters. It is worth mentioning that the Bayesian statistical analysis with non-informative priors was done on the GBD dataset, and then the information used to run the Bayesian analysis on the WHO dataset with informative priors came from the GBD dataset analysis. In other words, the information from two datasets was combined to give us better estimates about the pattern of prostate cancer death rates. In the Bayesian analysis of WHO data, the prior’s distributions regarding intercepts and slopes are assumed to be normal, and the mean and standard deviation are calculated using the GBD dataset.

In the first step, the Bayesian LGM with non-informative priors was applied to GBD data, and then the information was used to run the Bayesian LGM with informative priors on WHO data to estimate the overall growth. In the next step, we used Bayesian GMM to classify countries based on their growth patterns. In order to determine the optimal number of classes, the Bayesian GMM was first performed with two classes, and then the number of classes was increased in subsequent analyses until the best model fit was achieved. The best model with appropriate number of classes was determined by using the Entropy criterion which is using for assessing the quality of class membership classification [13]. The range of Entropy statistic varied from 0 to 1, where values greater than 0.80 are considered as high classification quality, and 0.60, and 0.40 are cut-off for indicating medium and low classification quality, respectively [13]. Due to the unknown class membership of each country, we were unable to use informative priors in the Bayesian GMM analysis. However, once the class membership of each country was determined, the growth trajectories of each class were separately estimated using the Bayesian LGM after the classification. Finally, the conditional Bayesian LGMs (models with covariates) were used for each class to estimate the effects of HDI, GNI, and LEB on the growth pattern of prostate cancer mortality rates over the study period. It is important to note that there were strong correlations between HDI, GNI, and LEB. As a result, to avoid possible multicollinearity between covariates, each covariate was estimated separately without making adjustments for other covariates.

To overcome the missing data problem, we used the "longitudinalData" package in the R software that provides functions to estimate missing data in longitudinal studies [22]. Also, Mplus 8.3 software was used for statistical analysis, and ArcGIS 10.8 was utilized to create a map illustrating the global geographic distribution of prostate cancer mortality rates.

## Results

**Fig 2:**
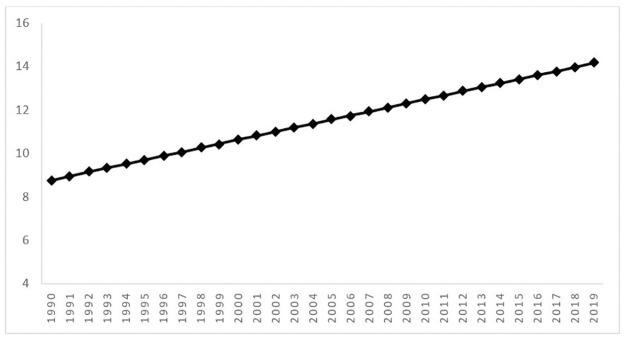
The estimated mean of the overall growth trajectory of prostate cancer mortality rates using unconditional Bayesian LGM.

The overall growth trajectory of prostate cancer mortality rates per 100,000 men from 1990 to 2019 in 109 countries was estimated using Bayesian LGM of WHO data with informative priors from GBD data. Table 1 shows that the intercept and the slope were equal to 8.77 and 0.19, respectively. Therefore, we can conclude that the overall mean of the prostate cancer death rate was 8.77 persons per 100,000 in the initial stage (in 1990), and the overall trend increased by 0.19 persons per 100,000 at every time point from 1990 to 2019. The estimated overall growth trajectory is shown in Fig 2.

**Table 1:** The estimated overall growth trajectory using the unconditional Bayesian LGM.

Latent Variable	Coefficient	Posterior SD	p-value
Intercept	8.774	0.682	< 0.001
Slope	0.186	0.020	< 0.001

Due to the inability of the Bayesian LGM model to account for the heterogeneity in the pattern of prostate cancer death rates across countries during the study period, a Bayesian GMM was employed to identify latent subgroups with distinct growth trajectories. The Bayesian GMM model with four classes yielded the highest Entropy statistic of 0.94, indicating a high-quality classification compared to other models with varying numbers of classes. The findings of the Bayesian GMM model with non-informative priors and four classes are presented in Table 2.

**Table 2:** The results of the Bayesian GMM model with non-informative priors.

Latent Class	Latent Variable	Coefficient	Posterior SD	p-value	Sample Size
1	Intercept	25.613	1.476	< 0.001	5 (4.6%)
	Slope	-0.057	0.055	0.298	
2	Intercept	2.476	2.690	< 0.001	63 (57.8%)
	Slope	0.147	0.168	< 0.001	
3	Intercept	8.152	2.769	< 0.001	11 (10.1%)
	Slope	0.513	0.172	< 0.001	
4	Intercept	13.668	0.553	< 0.001	30 (27.5%)
	Slope	0.026	0.024	0.276	

The intercept and the slope of the second class were 2.48 and 0.15, respectively, which indicates 57.8% of the study sample in the second class had the lowest death rates in the initial stage (it was equal to 2.48 persons per 100,000 men) and then increased linearly by 0.15 persons per 100,000 in each time point from 1990 to 2019. Although the slope of the first class was -0.06 and non-significant, its intercept was 25.61, which is very high in comparison to other classes. It can be concluded that the death rates of the first class was 25.61 persons per 100,000 in the beginning, and there was no significant pattern in the death rates of this class during the study period. In comparison to other classes, the third class had the highest increasing rate, which was equal to 0.51 person per 100,000 men. The last class, which comprised 30 countries, had an intercept of 13.67 and a non-significant slope. Hence, the initial death rate for this class was 13.67 per 100,000 men, and the trend did not change significantly over time. The Bayesian GMM analysis was done with non-informative priors, but after the class membership of each country was determined, the Bayesian LGMs with informative priors were conducted for each class. The results are shown in Table 3.

**Table 3:** The results of the Bayesian LGMs with informative priors for each subgroup.

Latent Class	Latent Variable	Coefficient	Posterior SD	p-value	Sample Size
1	Intercept	27.667	2.338	< 0.001	5 (4.6%)
	Slope	-0.098	0.170	0.546	
2	Intercept	3.26	0.335	< 0.001	63 (57.8%)
	Slope	0.209	0.022	< 0.001	
3	Intercept	10.158	1.423	< 0.001	11 (10.1%)
	Slope	0.691	0.114	< 0.001	
4	Intercept	15.366	0.869	< 0.001	30 (27.5%)
	Slope	0.081	0.041	0.030	

**Fig 3:**
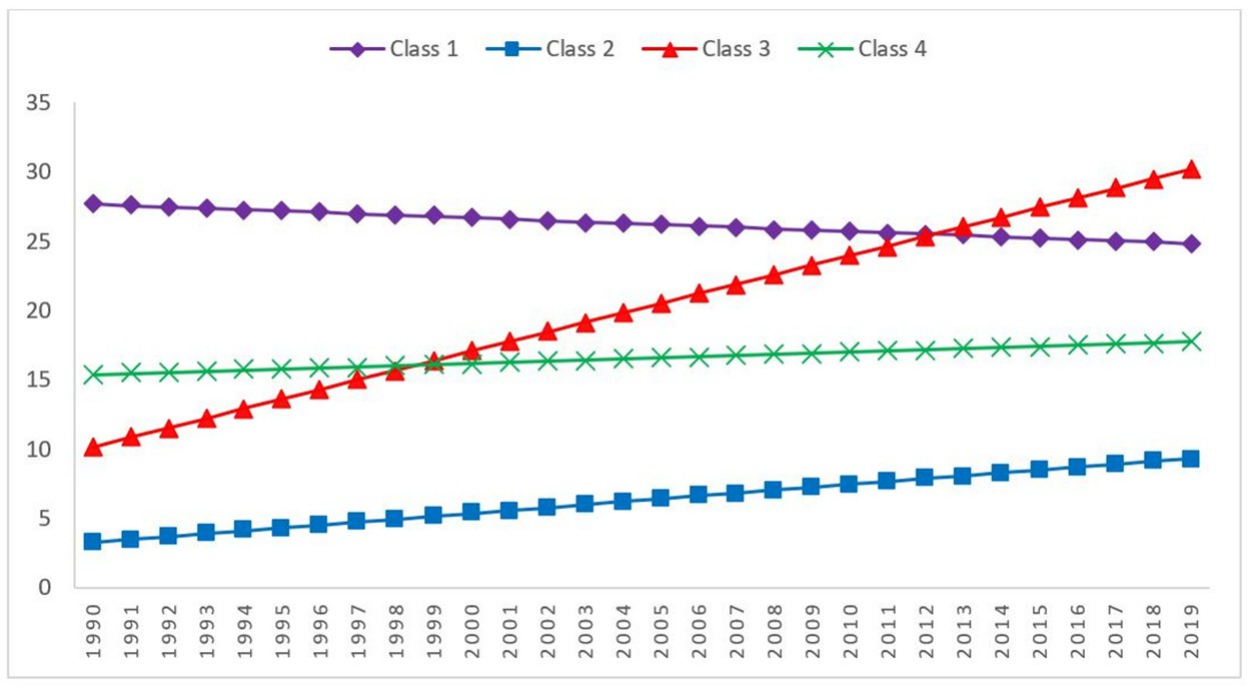
The estimated means of the four classes of unconditional Bayesian LGM with informative priors.

The results of the unconditional Bayesian LGMs with informative priors, as presented in Table 3, differ somewhat from those obtained from the Bayesian GMM with non-informative priors in Table 2. Notably, the intercept and slope estimates for the fourth class in Table 3 were 15.366 and 0.081, respectively, whereas in Table 2 they were 13.668 and 0.026. These findings suggest that using informative priors resulted in increased effects. The estimated growth trajectories of all classes using Bayesian LGM with informative priors are shown in Fig 3.

**Fig 4:**
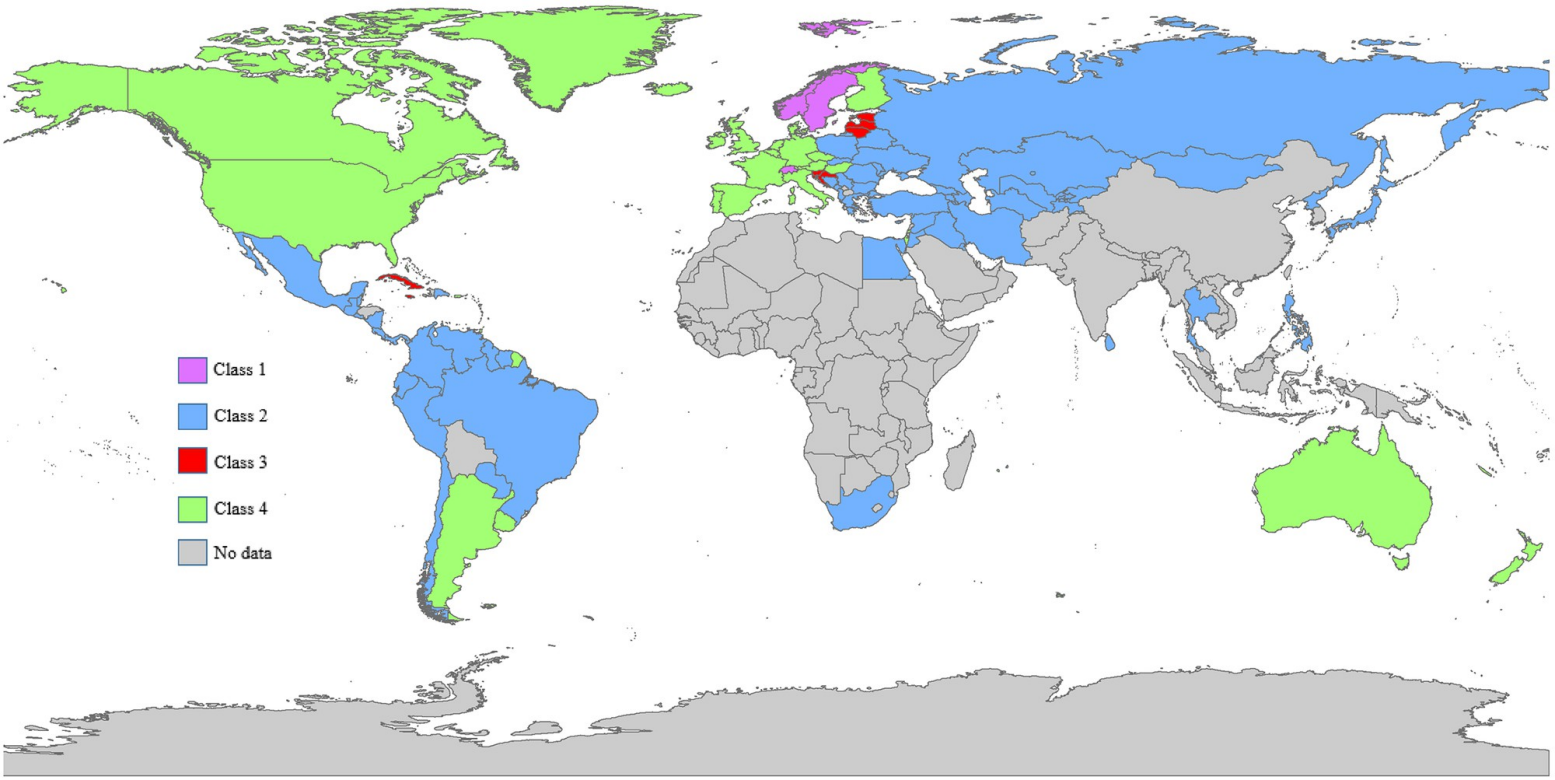
The geographic distribution of all the classes. The world map was created based on a map from http://www.naturalearthdata.com/, retrieved on July 9th, 2023. Please note that the map is for illustrative purposes only and is not identical to the original image.

The results of conditional Bayesian LGM models with informative priors are shown in Table 4. In conditional models, the quantity of the intercept and slope relies on the effect of the covariates, which makes it more difficult to interpret the effects. The intercept and slope of the total data were positively but not significantly affected by HDI. The effects of GNI on the intercept and slope of total data were 0.188 and -0.006, respectively, which means increasing GNI by $1000 is associated with increasing mortality rates by 0.188 persons per 100,000 men in the initial stage and decreasing the slope of death rates by 0.006 at each time point. Although GNI had a positive impact on the intercept of the overall data, it had a negative impact on the intercepts of the classes. While considering the entire dataset showed a positive association, focusing on classes revealed a negative association during the early stages of the study. Therefore, accounting for growth heterogeneity is crucial in drawing accurate conclusions about this relationship. The effects of Life Expectancy at Birth (LEB) on the intercept and slope of the entire set of data were 0.724 and -0.007, respectively. Therefore, a one-year increase in LEB can raise prostate cancer mortality rates by 0.724 per 100,000 men in the initial stage and lower the growth pattern’s slope by 0.007 per 100,000 men. For each class, LEB had different effects on intercepts, but they were all positive. While LEB had a significantly positive impact on the slope of the second class, it had a significantly negative impact on the slope of the fourth class. S1 Table shows the results of the Bayesian analysis of GBD data, which were used to set informative priors for the Bayesian analysis of WHO data. The geographic distribution of all classes across the world is depicted in Fig 4 and the class membership of each country showed in S2 Table.

**Table 4:** The results of the conditional Bayesian LGM models with informative priors.

Model	Coefficient	Class 1	Class 2	Class 3	Class 4	Total data
HDI	Intercept (Posterior SD)	23.607 (7.521) *	-5.363 (2.062) *	22.307 (7.261) *	13.735 (6.713) *	4.476 (4.565)
	Slope (Posterior SD)	0.773 (0.544)	-0.309 (0.151) *	1.591 (0.705) *	1.058 (0.496) *	0.025 (0.146)
	HDI on intercept (Posterior SD)	4.944 (0.094)	10.597 (2.872) *	-17.368 (9.397)	-0.003 (7.851)	3.592 (5.958)
	HDI on slope (Posterior SD)	- 1.105 (0.008)	0.634 (0.209) *	-1.353 (0.912)	-1.223 (0.580) *	0.156 (0.190)
GNI	Intercept (Posterior SD)	30.415 (3.923) *	3.083 (0.400) *	15.085 (2.191) *	15.582 (1.732) *	3.229 (0.909) *
	Slope (Posterior SD)	0.182 (0.308)	0.201 (0.028) *	0.749 (0.214) *	0.258 (0.103) *	0.299 (0.030) *
	GNI on intercept (Posterior SD)	- 0.087 (0.094)	- 0.026 (0.018)	-0.363 (0.121) *	-0.042 (0.043)	0.188 (0.032) *
	GNI on slope (Posterior SD)	- 0.007 (0.008)	- 0.002 (0.001)	-0.011 (0.012)	-0.006 (0.003) *	-0.006 (0.001) *
LEB	Intercept (Posterior SD)	4.989 (13.447) *	- 7.580 (3.545) *	- 0.987 (12.807)	6.738 (10.483)	-46.335 (7.027) *
	Slope (Posterior SD)	1.705 (1.003)	- 0.544 (0.254) *	1.451 (1.554)	2.374 (0.779) *	0.696 (0.253) *
	LEB on intercept (Posterior SD)	0.298 (0.179)	0.137 (0.049) *	0.134 (0.174)	0.091 (0.134)	0.724 (0.095) *
	LEB on slope (Posterior SD)	-0.024 (0.013)	0.010 (0.004) *	-0.012 (0.021)	-0.030 (0.010) *	- 0.007 (0.003) *

* Significant at 0.05 level

## Discussion

In this longitudinal study, the Bayesian LGM was utilized to estimate the overall mortality rate trend of prostate cancer in 109 countries from 1990 to 2019. The findings indicated an increasing overall growth trajectory of mortality rates across all 109 countries during the study period. However, the growth pattern of the prostate cancer mortality rate varied among different groups of countries. To explore the variation in growth trajectories of mortality rates among different countries, the Bayesian GMM was employed. The analysis identified four distinct latent subgroups with various intercepts and different growth patterns over time. The observed international variations in prostate cancer mortality rates could be associated with multiple factors, including ethnicity, life expectancy, income, education, the PSA screening program, health-care resources, HDI, and other risk factors such as obesity prevalence [5, 6, 10, 11]. In order to gain a deeper understanding of these variations and their underlying causes, it is crucial to examine the specifics of every subgroup that has been identified.

The first subgroup, which includes Switzerland, Norway, Sweden, and the two Caribbean countries of Martinique and Barbados, had the highest mortality rates initially and showed a relatively stable trend over time. Switzerland, Norway, and Sweden are among the countries with the highest standard of living in the world. However, they are also highly ranked in terms of mortality rates for men with prostate cancer [23, 24], which might be because of overdiagnosis of prostate cancer and high life expectancy in these countries. The high rates of prostate cancer mortality among Caribbean men are likely attributed to a combination of genetic predisposition and low socioeconomic status [25]. The second class, which included approximately 58% of the study sample, had the lowest mortality rate in the initial stage and an increasing trend over the study period. This class predominantly consists of developing countries in Asia, Eastern Europe, Africa, and some Latin American countries. The low mortality rates in these countries may be due to underreport, underdiagnosis, and a lower life expectancy in contrast to developed countries. Kimura discovered that the incidence of prostate cancer was higher among Asian immigrants in Western nations as compared to their native countries. She concluded that the lower rate of PSA screenings in Asian nations might play a role in the lower incidence of prostate cancer among Asian populations [7]. Increasing mortality rates in this class are thought to reflect westernization, including increased consumption of animal fat, high rates of obesity, and physical inactivity, especially in Asian countries [26]. The third class had a low initial mortality rate, but compared to the other classes, its prostate cancer mortality rate increased at the fastest rate. This class included some of the Caribbean islands and one island from eastern Africa (Seychelles), as well as some of the European countries such as Croatia, Slovenia, Lithuania, Latvia, and Estonia. A recent study has reported that prostate cancer accounts for 18%–47% of cancer-related deaths in the Caribbean region, making it the leading cause of male cancer deaths and the third leading cause of overall male deaths [27]. Clearly, prostate cancer is a major health issue in the Caribbean, particularly for the Black population, with high rates of occurrence and death. Research has shown that Black men have a higher risk of developing prostate cancer compared to men of other races. This is thought to be due to a combination of genetic, environmental, and lifestyle factors. [25, 27]. The class number four had the second highest prostate cancer mortality rate at the starting point of the study, followed by an almost stable pattern. This class mainly consisted of developed countries. It is noteworthy that almost 70% of the worldwide cases of prostate cancer are reported in developed countries, which could lead to high mortality rates in these countries [28]. In other words, prostate cancer incidence rates are higher in more developed countries because of the prostate cancer screening program, including the practice of PSA testing and subsequent biopsies, which induce a significant rise in reported prostate cancer incidence and mortality rates in these countries [28].

The conditional Bayesian LGM results indicated that the HDI had a positive but non-significant impact on both the intercept and slope of the total data. These results were inconsistent with the previous studies, that have reported a negative correlation between HDI and adjusted mortality rates [5, 29]. The impact of HDI on the different subgroups was found to vary, suggesting the presence of heterogeneity among countries. The GNI had a positive effect on the initial prostate cancer mortality rate, but it had a negative impact on the growth pattern. It is possible that the observed positive effect of GNI on the initial prostate cancer mortality rate is due to overdiagnosis of prostate cancer in high-income countries, resulting in increased detection. However, the negative effect of GNI on the growth pattern could be due to improved treatment of prostate cancer, leading to a decrease in mortality rates over time in these countries. Previous studies have shown that there is a global variation in the burden of prostate cancer with respect to GDP, where some studies have reported a negative correlation while others have reported a positive one [4, 30, 31]. The effect of LEB on the total data of prostate cancer mortality rates was positive in the initial but negative on the growth trajectory. This suggests that countries with high life expectancy had high prostate cancer mortality rates, but most of these countries, which are developed, improved their treatment and decreased their prostate cancer death rates. In a cross-sectional study conducted in 2012 on the prostate cancer dataset of Asian countries, there was a non-significant positive correlation of 0.125 between the standardized mortality rate and life expectancy at birth [32], on the other hand, a negative correlation between life expectancy at birth and standardized mortality rate has been reported for countries worldwide in 2012 [5].

### Strengths and limitations

The main strength of this study lies in its application of the Bayesian approach to integrate two data sources, (WHO and GBD) in order to estimate prostate cancer mortality rates. Furthermore, this study addressed the heterogeneity in prostate cancer mortality rates through the Bayesian GMM approach and estimated the effects of HDI, GNI, and LEB for each subgroup. Nevertheless, it is important to acknowledge several limitations in our study. The quality and accessibility of the original data in the GBD platform varied between countries, and some data were indirectly estimated using modeling techniques. Another important limitation is the possibility of underreporting of prostate cancer data by the WHO in countries with limited resources. This could lead to an underestimation of the actual mortality rates in these regions, which may impact the accuracy of the findings. Finally, it is essential to acknowledge that the covariates we analyzed exhibited a high degree of correlation. This could lead to multicollinearity, making it challenging to account for the impact of each individual covariate. Thus, caution is warranted in interpreting the results, as there could be other confounding factors that influence the association. It is crucial to keep in mind that a significant association does not necessarily imply causation. Furthermore, there are several variables that we did not consider in our statistical analysis, such as race and ethnicity, percentage of immigrant population, and smoking rate. These factors represent additional limitations of our study.

### Conclusion

The mortality rate due to prostate cancer has increased globally, but the trend varies between countries. Northern European countries and those in the Caribbean region have recorded the highest mortality rates. Developed countries have consistently high mortality rates, while less developed countries show a rising pattern despite having lower mortality rates. Moreover, the impact of HDI, GNI, and LEB on mortality rates differs considerably among the four subgroups, signifying a need for further investigation to identify the causal factors.

## Supporting information

S1 Table The results of Bayesian LGM of GBD data with non-informative priors. * Significant at 0.05 level.
(PDF)

S2 Table The class membership determined by Bayesian Growth Mixture Model. (PDF)
