# Supplementary material for: Bayesian clustering of 109 worldwide countries according to the trend of prostate cancer mortality rates from 1990 to 2019
Source: PLoS One. 2023 Aug 14;18(8):e0290110. doi: 10.1371/journal.pone.0290110 (PMC10424856; doi:10.1371/journal.pone.0290110)
Supplement: S1 Table — * Significant at 0.05 level. (PDF) [file pone.0290110.s001.pdf]

Model	Coefficient	Class 1	Class 2	Class 3	Class 4	Total data
HDI	Intercept (Posterior SD)	27.510 (13.955)	-3.278 (5.888)	26.157 (12.257) *	25.978 (11.955) *	56.087 (11.367) *
	Slope (Posterior SD)	0.898 (0.874)	-0.303 (0.343)	2.082 (1.109)	1.392 (0.897)	-0.245 (0.423)
	HDI on intercept (Posterior SD)	23.461 (16.773)	16.142 (8.230)	-1.301 (15.659)	7.995 (13.964)	-23.995 (8.711) *
	HDI on slope (Posterior SD)	-1.265 (1.106)	0.892 (0.477)	-1.392 (1.427)	-1.409 (1.050)	0.542 (0.324)
GNI	Intercept (Posterior SD)	25.139 (15.813)	8.130 (1.080) *	36.634 (7.578) *	31.931 (4.635) *	9.512 (2.395) *
	Slope (Posterior SD)	0.330 (0.467)	0.349 (0.057) *	1.200 (0.429) *	0.500 (0.207) *	0.512 (0.063) *
	GNI on intercept (Posterior SD)	0.580 (0.451)	0.003 (0.052)	-0.714 (0.428)	0.023 (0.118)	0.402 (0.085) *
	GNI on slope (Posterior SD)	-0.011 (0.011)	-0.001 (0.003)	-0.011 (0.024)	-0.008 (0.005)	-0.007 (0.002) *
LEB	Intercept (Posterior SD)	4.937 (19.650)	-3.325 (10.101)	10.805 (19.548)	13.581 (18.384)	-43.772 (15.489) *
	Slope (Posterior SD)	1.849 (1.593)	-0.645 (0.579)	1.476 (1.839)	2.991 (1.386) *	0.877 (0.605)
	LEB on intercept (Posterior SD)	0.652 (0.305) *	0.162 (0.141)	0.200 (0.270)	0.247 (0.236)	0.849 (0.210) *
	LEB on slope (Posterior SD)	-0.026 (0.021)	0.014 (0.008)	-0.006 (0.025)	-0.036 (0.018)	-0.007 (0.008)
Unconditional	Intercept (Posterior SD)	40.444 (12.787)	8.195 (0.729) *	25.117 (3.656)	28.519 (1.801) *	18.455 (1.588) *
	Slope (Posterior SD)	-0.049 (0.286)	0.332 (0.038) *	1.021 (0.166)	0.194 (0.067) *	0.357 (0.039) *
