# Supplementary material for: Bayesian clustering of 109 worldwide countries according to the trend of prostate cancer mortality rates from 1990 to 2019
Source: PLoS One. 2023 Aug 14;18(8):e0290110. doi: 10.1371/journal.pone.0290110 (PMC10424856; doi:10.1371/journal.pone.0290110)
Supplement: S2 Table — (PDF) [file pone.0290110.s002.pdf]

Class 1	Switzerland, Norway, Sweden, Martinique, Barbados
Class 2	Iraq, Turkmenistan, Uzbekistan, Maldives, Tajikistan, Sri Lanka, Egypt, Syrian Arab Republic, Kuwait, Mongolia, Jordan, Bahrain, Thailand, Kyrgyzstan, Iran, Brunei Darussalam, Azerbaijan, Philippines, Kazakhstan, Republic of Korea, Mayotte, Nicaragua, El Salvador, Guatemala, Fiji, Turkey, Singapore, Hong Kong, Albania, Peru, Paraguay, Republic of Moldova, Mauritius, Georgia, Mexico, South Africa, Ecuador, Armenia, Colombia, Brazil, Suriname, Russian Federation, Ukraine, Belize, North Macedonia, Reunion, Dominican Republic, Venezuela, Bosnia and Herzegovina, Costa Rica, Japan, Belarus, Panama, Montenegro, Guyana, Cyprus, Romania, Chile, Poland, Slovakia, Bulgaria , Serbia, Greece
Class 3	Croatia, Lithuania, Seychelles, Latvia, Estonia, Slovenia, Saint Lucia, Jamaica, Cuba, Saint Vincent and the Grenadines, Grenada
Class 4	France, Luxembourg, United States of America, Canada, Italy, Ireland, Hungary, Czechia, Spain, Germany, Bahamas, Australia, New Zealand, Austria, Puerto Rico, Belgium, French Guiana, Israel, Finland, Malta, Argentina, Netherlands, Iceland, Portugal, Uruguay, United Kingdom, Trinidad and Tobago, Denmark, Guadeloupe, Antigua and Barbuda
